# Evolutionary dynamics and functional divergence of the UDP-glycosyltransferases gene family revealed by a pangenome-wide analysis in tomato

**DOI:** 10.1093/hr/uhaf204

**Published:** 2025-07-21

**Authors:** Miaomiao Huang, Peng Zheng, Ning Li, Qionglin Chen, Yan Liu, Benliang Huang, Xiaoyuan Tao, Jingyin Yu, Shengchun Xu

**Affiliations:** Xianghu Laboratory, Hangzhou 311231, P.R. China; Xianghu Laboratory, Hangzhou 311231, P.R. China; Biological Breeding Laboratory, Xinjiang Uygur Autonomous Region Academy of Agricultural Sciences, Urumqi, China; Xianghu Laboratory, Hangzhou 311231, P.R. China; Xianghu Laboratory, Hangzhou 311231, P.R. China; Xianghu Laboratory, Hangzhou 311231, P.R. China; Xianghu Laboratory, Hangzhou 311231, P.R. China; Xianghu Laboratory, Hangzhou 311231, P.R. China; Xianghu Laboratory, Hangzhou 311231, P.R. China; Institute of Digital Agriculture, Zhejiang Academy of Agricultural Science, Hangzhou, China

## Abstract

UDP-dependent glycosyltransferases (UGTs) play a critical role in producing glycosylated metabolites that mediate plant–environment interactions. Recent studies have examined the role of *UGT* genes across various plant genomes. However, the evolutionary history and functional divergence of the *UGT* pan-gene family in the genus *Solanum* have not yet been explored. This study integrated data from 61 tomatoes and 9 representative genomes, ranging from algae to angiosperms, to identify 12 073 genes. The phylogeny of the UGT gene family reveals a clear evolutionary trajectory from unicellular algae to ferns, mosses, gymnosperms, and angiosperms. The study identified a significant number of tomato-specific *UGT* genes and explored the expansions of *UGT73* and *UGT85* subfamilies. The entire *UGT* genes (10 769) in tomato were classified into 118 orthologous gene groups, including 58 core, 31 softcore, 10 dispensable, 19 private orthologous gene groups, and the core groups contained 7811 genes, representing 72.53% of the total *UGT* genes. Analysis of gene family expansion revealed that whole-genome triplication and tandem duplication events play significant roles in the expansion of the *UGT* gene family. Selection pressure analysis revealed that the *UGT* genes experienced purifying selection in the genus *Solanum*. Additionally, expression profiles of some *UGT* genes in different tissues demonstrated expression divergence of multicopy genes across different *UGT* subfamilies due to the increase in gene dosage. Subcellular localization prediction revealed that most genes are localized in the chloroplast. These findings provide critical insights into the evolution and function of the *UGT* genes in tomato, laying a foundation for further exploration in adaptive evolution.

**Abbreviations**
HMM: Hidden Markov Model
OGG: orthologous gene groups
PAVs: presence–absence variations
SLC: the cherry-sized tomato
SLL: the large-fruited modern tomato
SP: the blueberry-sized tomato
TD: tandem duplication
UGT: UDP-dependent glycosyltransferase
WDR: the wild distant relatives of tomatoes
WGT: whole-genome triplication

## Introduction

Glycosylation is a fundamental cellular modification present in all living organisms, where carbohydrate molecules are added to other molecules. In plants, glycosylated compounds are essential for a range of processes, including hormone regulation, pollination, UV protection, defense mechanisms, and the detoxification of foreign substances [1, 2]. These glycosylation reactions are facilitated by glycosyltransferase enzymes (GTs), which exhibit considerable diversity, originating from multiple evolutionary lineages and forming a large multigene family [3, 4]. According to the latest update from CAZy (http://www.cazy.org/GlycosylTransferases.html), GTs from different species are categorized into 137 families. Among these, Family-1 GTs are the most prevalent glycosyltransferases in the plant kingdom. UDP-dependent glycosyltransferases (UGTs), which are part of the largest family (Family 1), demonstrate unique yet overlapping substrate specificities [5].

UDP-glycosyltransferases (UGTs) are enzymes that catalyze the covalent addition of sugar molecules to a wide range of lipophilic compounds. These enzymes not only glycosylate various acceptor molecules such as anthocyanidins, flavonoids, saponins, sterols, terpenoids, phenylpropanoids, and plant hormones, but also detoxify and deactivate xenobiotics, playing a crucial role in plant–pathogen interactions [6, 7]. For instance, in wheat, UGTs contribute to detoxification and enhance resistance to *Fusarium* head blight (FHB) by converting deoxynivalenol (DON) into DON-3-glucoside (D3G) [8]. The UDP-glycosyltransferase gene family is highly diverse, comprising ~200 identified subfamilies: subfamilies 1–50 are found in animals, 51–70 in yeast, 71–100 in plants, and 101–200 in bacteria [5]. Different *UGT* subfamilies are associated with specific functions. For example, overexpressing the gene of *UGT85* subfamily in transgenic tobacco has been shown to enhance salt tolerance [9]. Additionally, *UGT73* subfamily gene in *Arabidopsis thaliana* responds to pathogens and is essential for resisting biotic stresses [10]. The plant *UGT* gene family is classified within 30 subfamilies, spanning *UGT71* to *UGT100* [11]. In *A. thaliana*, there are 122 genes distributed across 21 subfamilies, providing a valuable reference for research as a model plant. Extensive research has identified and classified *UGT* in various plants, including *A. thaliana*, *Linum usitatissimum*, *Triticum aestivum*, *Zea mays*, *Gossypium hirsutum*, and *Prunus persica* [12–15]. The variation in gene family size and subfamily conservation across different species contains key information that may help us understand plant evolutionary history and environmental adaptation [7].

Reference genome availability has expanded exponentially in the past two decades [16]. The reference genome only represents the genetic content of a single individual and thus cannot reflect the genetic diversity across different strains, particularly presence–absence variations (PAVs) [17–19]. Based on orthologous relationships among different species, a pan-gene family can be constructed to systematically investigate the PAV patterns across different subfamilies. According to gene conservation, the pan-gene family can be further classified into core genes (strictly conserved) and variable/dispensable genes (absent in at least one species). This classification is biologically significant for understanding gene family evolution [20, 21]. Using tomato pangenome data, this study aims to overcome the limitations of single reference genomes and comprehensively characterize the evolutionary state of *UGT* gene families in the genus *Solanum*. The available genomes, *Physcomitrium patens* and *Adiantum capillus-veneris*, were recognized as the representatives of bryophytes and pteridophytes, with the genomes of *Ginkgo biloba* and *Cycas panzhihuaensis* representing gymnosperms. In angiosperms, the most basal extant flowering plant (*Amborella trichopoda*) [22], a true diploid without a recent genome duplication after the angiosperm whole-genome triplication (WGT)-γ event (*Vitis vinifera*) [23], the model species (*A. thaliana*) [24], and a close relative to tomato in the *Solanum* genus (*Solanum tuberosum*) [25] were included for the downstream analysis.

Tomato (*Solanum lycopersicum* L.) is one of the highest-yielding vegetable/fruit crops globally, exhibiting significant genetic diversity shaped by domestication and breeding [21]. Wild tomatoes exhibit diverse fruit colors (e.g. red, green) and significant variation in flavor and nutritional traits [26]. These wild relatives provide the blueprint for the evolutionary process of domesticated tomato, and their genetic diversity laid the foundation for the selective breeding of modern tomato varieties [27]. Through domestication and selection, the fruit morphology and functions of wild tomato gradually changed, eventually leading to the formation of diverse tomato varieties. The domestication of tomato can be divided into two main stages: from the blueberry-sized *Solanum pimpinellifolium* (SP) to the cherry-sized *S. lycopersicum* var. *cerasiforme* (SLC), and then to the large-fruited modern tomato *S. lycopersicum* var. *lycopersicum* (SLL) [26]. SP is the ancestor of domesticated tomato [28], belonging to the wild relative branch of tomato, with mature fruits in red or green, exhibiting significant differences in flavor and nutritional traits [29]. After initial domestication, the cherry-sized SLC was formed, which is an evolutionary intermediate between SP and modern large-fruited tomato. Ultimately, SLC was further improved and developed into the modern large-fruited SLL, including early cultivated varieties (heirloom varieties), modern domesticated varieties, and contemporary market varieties (fresh market) [30, 31]. Based on 61 tomato genomes, we constructed a pan-gene dataset for the tomato *UGT* gene family. By scanning all the genome assemblies, homologous gene groups were identified and characterized for gene family profiling. Using the concepts of core group and dispensable group, new insights into the evolution of the *UGT* gene family were provided. Further, through phylogenetic analysis, the evolutionary history of the *UGT* gene family was explored; WGT and tandem duplication (TD), selection pressure, and expression analysis were performed to reveal the unique evolutionary process of the tomato *UGT* gene family.

## Results

### Identification of the *UGT* gene family in the genomes

**Figure 1:**
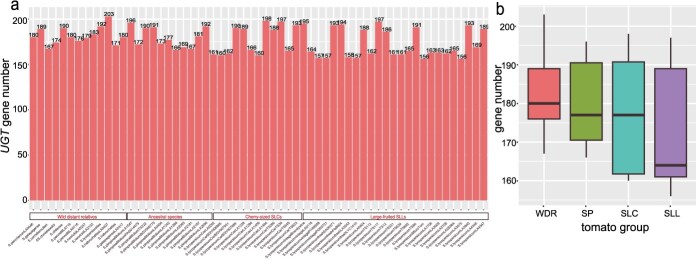
The number of *UGT* genes in tomatoes. (a) Each tomato species is labeled according to its group below the bars, while the numbers on the bars represent the number of UGT genes present in each species. (b) The *UGT* gene number across the four tomato groups, including WDR, SP, SLC, and SLL.

A total of 61 tomato genomes, including 13 distant wild relatives (WDR), 11 blueberry-sized tomatoes, 12 cherry-sized tomatoes, and 25 large-fruited tomatoes, were collected from public databases to implement the identification of *UGT* genes in the genus *Solanum* (Table S1). Except, the representative species from unicellular algae (*Chlorella variabilis*), bryophytes (*P. patens*), pteridophytes (*A. capillus-veneris*), gymnosperms (*G. biloba* and *C. panzhihuaensis*), to higher angiosperms (*A. trichopoda*, *V. vinifera*, *A. thaliana*, and *S. tuberosum*) were included in the analysis to trace the origin and evolution of *UGT* gene family. With the characteristics of conserved domains of *UGT* proteins, a total of 12 073 *UGT* genes were identified across 70 plant genomes and 10 769 *UGT* genes in 61 tomato genomes (Table S2). These 10 769 *UGT* genes belong to 21 subfamilies (Fig. 1a). The tomato species with the highest number of *UGT* genes was WDR cv. LA0407, with a total of 203 genes, whereas SLL cv. MicroTom has the lowest number, with 156 *UGT* genes. In terms of gene numbers, the WDR group has the highest average number, reaching 182 genes, followed by the SP group with an average of 180 *UGT* genes. The SLL group has the lowest average number, with 171 genes, while the SLC group has an average of 177 *UGT* genes. These results indicate a general decline in *UGT* gene number from ancestral tomato species to modern cultivated varieties (Fig. 1b). The oldest algae have the fewest *UGT* genes, with only 6, followed by ferns with 26 genes. Notably, the number of *UGT* genes shows a significant increase in mosses, reaching 148, while the numbers in the two gymnosperms were nearly identical (163 and 164, respectively). The number of *UGT* genes significantly increases in angiosperms, with the higher numbers observed in *V. vinifera* (246) and *S. tuberosum* (299), while the number in *A. thaliana* (138) was similar to previously reported numbers [32]. Based on the number of *UGT* genes in different species, the results indicate that *UGTs* were widely distributed across the plant kingdom and have undergone significant expansion (*P* = 0.01533). There was a general trend of increasing numbers of *UGT* per genome during plant evolution.

### Phylogenetic analysis of *UGT* genes

**Figure 2:**
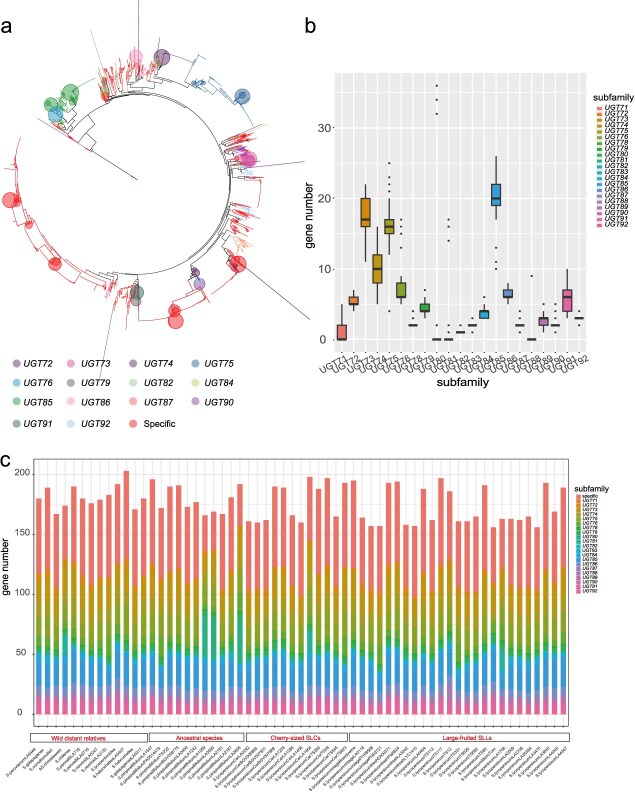
Phylogenetic tree of *UGT* genes across 70 plant genomes. (a) The phylogenetic tree shows the relationships between different *UGT* subfamily members, with each subfamily represented by a unique color that corresponds to the labels in the tree. The branch lengths and distribution reflect the evolutionary distances between subfamilies. The branches of the same subfamily were clustered into a circle, with the circle size proportional to the number of members. (b) The distribution of gene number among different *UGT* subfamilies in tomato, highlighting the variation in the number of members within each subfamily. (c) The distribution of different *UGT* subfamilies across 61 tomato species.

The entire protein sequences from 61 tomato genomes and 9 genomes were organized to construct a phylogenetic tree (Table S3) of the *UGT* gene family with the Q.plant R7 model. Three *UGT* genes from *C. variabilis* genome were clustered together, indicating independent evolution in unicellular algae, and the rest of the *UGT* genes were clustered into a separate group. The phylogenetic tree of the entire *UGT* genes was rooted with sequences from unicellular algae, which represent the basal clade in the tree across 70 genomes. The branches distant from the root were mainly specific to particular species, with the tomato-specific *UGT* genes representing the independent evolution within the *Solanum* genus. The phylogenetic tree demonstrated a clear evolutionary trajectory, with *UGT* families evolving sequentially from unicellular algae to ferns, gymnosperms, and finally to angiosperms. This pattern reflects the gradual evolution of the *UGT* family across lineages, with each subfamily evolving in a manner that mirrors the evolutionary history of *UGT* families (Fig. 2a).

Apart from the tomato-specific *UGT* genes, the subfamily with the highest proportion in each tomato *UGT* family were the UGT85 subfamily (average of 11.37%) and *UGT73* subfamily (average of 9.90%). The other two *UGT* subfamilies with at least 10 members were *UGT74* (5–16) and *UGT75* (4–25) (Fig. 2b). *UGT80* was detected only in some tomatoes, such as SP (LA1269, LA2093, LA2187, and LA2656), SLC (BGV007989), and SLL (LA3242, LA1706, and TS12). The *UGT82* subfamily has only one gene in each tomato species, indicating absolute conservation of this subfamily in the genus *Solanum*. The remaining subfamilies were relatively evenly distributed across different tomatoes, including *UGT72* (4–7), *UGT76* (5–17), *UGT78* (2–4), *UGT79* (3–7), *UGT83* (1–3), *UGT84* (3–6), *UGT86* (5–8), *UGT87* (1–4), *UGT89* (1–5), *UGT90* (1–5), and *UGT91* (3–10). Several tomato-specific *UGT* genes were identified (Fig. 2c), and it was found that the number of species-specific *UGT* genes in tomato was relatively stable, at around 60. The stable number of tomato-specific genes in each genome suggested that these genes might be conserved in the tomato genomes and likely played an important role in tomato evolution. The phylogenetic tree, such as *UGT90*, *UGT89*, *UGT92*, *UGT72*, and *UGT73* subfamilies, exhibit clustering with sequences from *V. vinifera*, *A. thaliana*, and other angiosperms, suggesting their divergence more recently within these lineages. In contrast, other subfamilies, including *UGT75*, *UGT87*, *UGT86*, *UGT82*, *UGT83*, as well as *UGT85* and *UGT78*, cluster with the more ancient gymnosperms, indicating their older evolutionary origin and highlighting their functional characteristics that were likely conserved since the divergence of gymnosperms.

### *UGT* pan-gene family

**Figure 3:**
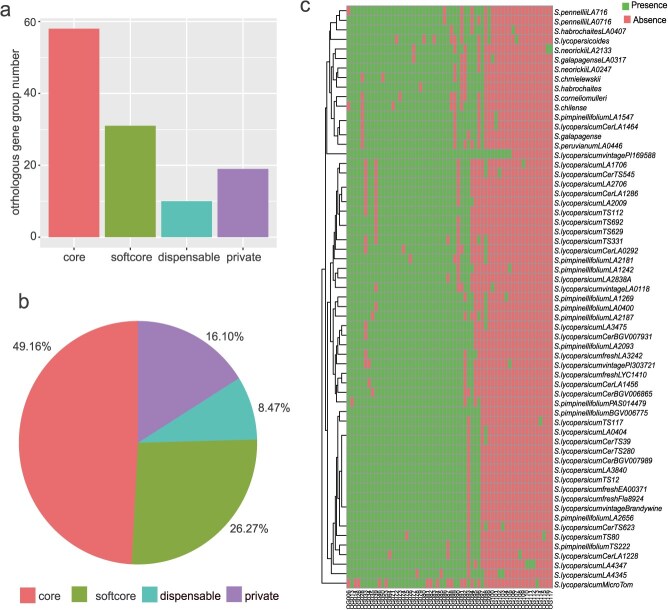
Orthologous gene groups of 61 tomatoes. (a) Distribution of orthologous gene groups. The bar chart shows the number of genes categorized as core, softcore, dispensable, and private OGGs within the UGT pan-gene family. (b) Proportional representation of OGGs in genomes. The pie chart illustrates the percentages of genes categorized as core, softcore, dispensable, and private OGGs, highlighting their relative abundance within the genomes. (c) Heatmap representing the distribution of presence/absence variation (PAV) across OGGs.

The pangenome, by integrating the genome sequences of different varieties, can present genetic information within a species in a more comprehensive and reference-free manner compared to a single reference genome. So, the members of the *UGT* subfamily in single reference genome do not represent the feature of the *UGT* subfamily within the genus *Solanum*. The entire *UGT* subfamily across 61 tomato genomes comprised the *UGT* pan-gene family. To investigate the conservation of *UGT* genes in the tomato UGT pan-gene family, the entire *UGT* gene family (10 769) was classified into 118 orthologous gene groups (OGGs) (Table S4). A total of 58 core OGGs (present in all 61 varieties), 31 softcore OGGs (conserved in 60 varieties), 10 dispensable OGGs (2–60 varieties), and 19 private OGGs (1 variety) were identified (Fig. 3a). The 58 core OGGs containing 5811 *UGT* genes account for 49.2% of total *UGT* genes in the genus *Solanum*, while private OGGs make up only 16.1% (Fig. 3b).

Among the core orthologous gene groups, excluding tomato-specific genes, the relatively abundant subfamilies among the 7811 *UGT* genes were *UGT85* (13.74%) and *UGT73* (10.92%). The *UGT80* and *UGT81* subfamilies were absent in the core OGGs. In contrast, all 58 members of the *UGT71* subfamily were present in the core pan-gene set. Notably, *UGT73* subfamily (7.06%) was the subfamily with a relatively high number of *UGT* genes among the softcore OGGs. However, *UGT74* subfamily (7.03%) has overtaken *UGT85* subfamily (4.68%) to rank second, while *UGT75* subfamily (4.61%) were another highly represented subfamily in softcore OGGs. Private OGGs mainly focused on the *UGT74*, *UGT75*, *UGT79*, *UGT80*, *UGT81*, *UGT85*, *UGT86*, *UGT90*, and *UGT91* subfamilies, indicating the specificity of these subfamilies. Among the 60 presence/absence variation (PAV) OGGs (Fig. 3c), the *UGT73* and *UGT85* subfamilies were relatively more numerous, indicating that these two subfamilies have undergone functional divergence (Table S4). However, the subfamilies *UGT71*, *UGT76*, *UGT78*, *UGT80*, *UGT82*, *UGT84*, and *UGT87* were absent from the PAV analysis (Table 1).

**Table 1:** . Distribution of 118 OGGs among *UGT* subfamilies in different groups.

	Core	Dispensable	Private	Softcore
*UGT71*	✓	✗	✗	✗
*UGT72*	✓	✗	✗	✓
*UGT73*	✓	✓	✗	✓
*UGT74*	✓	✗	✓	✓
*UGT75*	✓	✓	✓	✓
*UGT76*	✓	✗	✓	✗
*UGT78*	✓	✗	✗	✓
*UGT79*	✓	✗	✓	✓
*UGT80*	✗	✓	✓	✓
*UGT81*	✗	✓	✓	✓
*UGT82*	✓	✗	✗	✗
*UGT83*	✓	✗	✗	✓
*UGT84*	✓	✓	✗	✗
*UGT85*	✓	✓	✓	✓
*UGT86*	✓	✓	✓	✗
*UGT87*	✓	✓	✗	✗
*UGT88*	✗	✓	✗	✓
*UGT89*	✓	✗	✗	✓
*UGT90*	✓	✗	✓	✓
*UGT91*	✓	✓	✓	✗
*UGT92*	✓	✗	✗	✓

A check mark indicates presence, while a cross mark indicates absence.

In the presence/absence variation analysis, most orthologous gene groups did not exhibit a consistent pattern of distribution across different tomato groups along the domestication trajectory from wild to cultivated accessions (WDR, SP, SLC, SLL) (Table S5) except for six OGGs. Specifically, 3 OGGs showed a gradually rising frequency from wild to cultivated accessions: OG0000028 (62%, 91%, 92%, 100%), OG0000089 (38%, 82%, 92%, 100%), and OG0000092 (46%, 73%, 83%, 84%). In contrast, the other 3 OGGs showed a gradual decrease in frequency across the same groups: OG0000039 (100%, 91%, 83%, 68%), OG0000087 (100%, 91%, 92%, 88%), and OG0000090 (100%, 82%, 75%, 68%). These 6 OGGs were identified as tomato specific, suggesting that these gene sets may have played important roles during the domestication process of tomato.

### Gene duplication events in tomato

Gene duplication is an important mechanism that promotes the increase in gene copies in the gene family. In this part, we investigated the influence of two main mechanisms, whole-genome triplication (WGT) and tandem duplication (TD) events, on the expansion of the *UGT* gene family in the genus *Solanum*. A total of 3522 *UGT* genes representing 32.7% of total *UGT* genes were shown to experience TD events; these *UGT* genes were distributed into 1106 tandem arrays arranged from 6 to 39 arrays in tomato species. In the WDR group, the number of *UGT* genes that underwent TD events account for 32.78%–57.81%, whereas in the SP group, the proportion of gene numbers ranged from 21.56% to 38.02%. In the SLC group, the highest proportion was 47.98%, while the lowest was 22.29%. In the SLL group, which contained more members, the proportions were 19.87% and 44.38%, respectively. These results indicated that the WDR group had the highest proportion of genes undergoing TD events, while the proportion of this showed a increasing trend from 12.69% to 36.93% (Fig. S1). Among four tomato groups, the tomato-specific *UGT* genes experienced the strongest influence of TD events. The subfamilies with the second highest number of *UGT* genes influenced via TD events varied among groups, being UGT73 in SP, and *UGT85* in SLC and SLL. These findings suggest that the expansion of *UGT* gene families in tomatoes was largely driven by TD events, which play a crucial role in shaping the diversity and specialization of *UGT* genes across different tomato groups.

In this study, we also analyzed the effect of the WGT event on the expansion of *UGT* genes in tomatoes. Among the 61 tomato varieties, the average number of *UGT* genes in SP tomatoes was 180, with *UGT* genes undergoing WGT event accounting for 14.06%–19.28%. In the 12 SLCs, SLC (LA1456) exhibited the highest number of *UGT* genes undergoing WGT event, with 31 *UGT* genes, representing 25%. In the SLL group, which consists of 25 members, the highest and lowest proportions of WGT event were 21.15% and 12.69%, corresponding to 33 and 25 *UGT* genes, respectively. In the 61 tomato species, a total of 3674 specific genes were identified, with 342 of these genes having undergone TD events (Fig. S1). Additionally, 280 genes were found to have undergone a WGT event. This indicated that both TD and WGT events have contributed significantly to the expansion of unique tomato gene subfamilies, with a substantial number of *UGT* genes being specific to the tomato species.

As shown in the phylogenetic tree of the *UGT* gene family (Fig. S2), tandem duplication (TD) and whole-genome triplication (WGT) have significantly contributed to the expansion and phylogenetic structure of the gene family. TD events are highly concentrated in subfamilies such as *UGT85*, *UGT73*, *UGT76*, and *UGT91* subfamily, forming distinct clustered branches, indicating extensive TD within these clades. In contrast, WGT-derived genes are primarily distributed in subfamilies such as *UGT72*, *UGT89*, and *UGT92* subfamily, with a more scattered topology. The lower part of the tree, which lacks subfamily annotations, is mostly composed of tomato-specific sequences, where TD-derived and non-duplicated genes account for the majority. Overall, the distribution patterns of TD and WGT across different subfamilies have markedly shaped the phylogenetic structure of the *UGT* gene family.

### Selection pressures on *UGT* orthologs in tomatoes

Selection pressure reveals the evolutionary forces shaping genetic variation, driving adaptation, maintaining functional constraints, or promoting divergence among species. It is typically assessed by the ratio of nonsynonymous (*K*a) to synonymous (*K*s) substitution between *V. vinifera* and individual tomato genomes. Through collinear analysis between *V. vinifera* and individual tomato genomes, a total of 2369 *UGT* gene pairs were identified. Analysis of the *K*a/*K*s ratios of orthologous gene pairs indicated that a large proportion of *UGT* genes across the 61 tomato genomes experienced purifying selection (*K*a/*K*s < 1). This trend reflects the conservation of *UGT* genes during evolution, contributing to the maintenance of their function and adaptability. Although the *K*a/*K*s values of both groups are below 1, indicating that they are generally subject to purifying selection, the average *K*a/*K*s value of the WDR group (0.219) is higher than that of the cultivated SLL group (0.194), suggesting relatively weaker selective constraints (Fig. S3). This pattern may reflect a broader spectrum of functional variation retained in wild species, potentially contributing to their greater adaptive capacity in natural environments.

**Figure 4:**
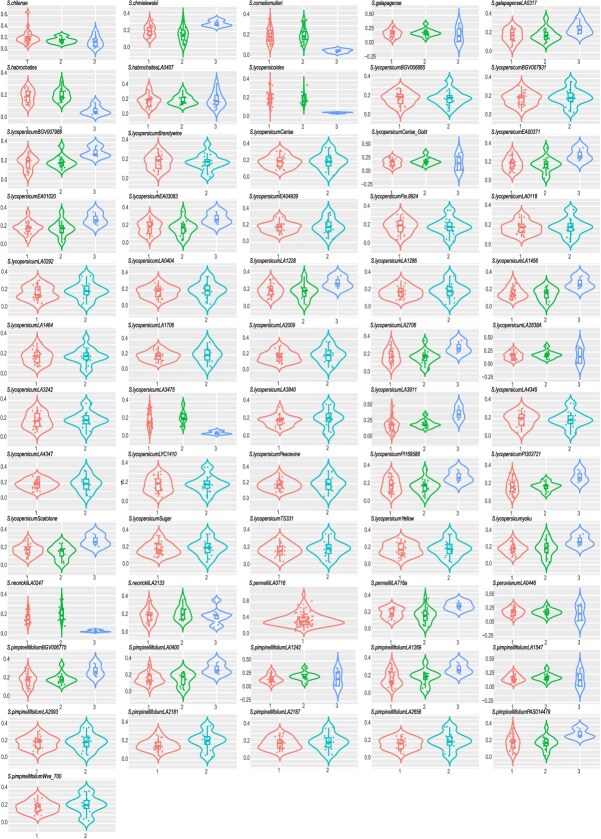
Retention patterns of gene pairs across 61 tomato species following the whole-genome triplication (WGT) event in the *Solanum* lineage are shown with violin plots displaying selection pressure (*K*a/*K*s values) for gene pairs categorized by copy number.

A whole-genome triplication (WGT) event occurred in the *Solanum* lineage, leading to three-, two-, and one-gene copies retained, characterized by extensive gene expansion and subsequent loss due to the gene dosage balance hypothesis [33]. Comparative analysis between *V. vinifera* and individual tomato genomes revealed selection pressures on gene pairs categorized by the copy numbers: three-, two-, and one-gene copy groups (Fig. 4). Among the 61 tomato species, certain individuals exhibit notable differences in selection pressure across different copy number groups. For example, in WDR cv. LA1028, the average ratios in the group with three-gene copies were higher than that in the other two groups; this pattern was also observed in SP cv. PAS01447, WDR cv. LA716a, and SP cv. LA1269. Overall, in these four species, the selection pressure on three-copy genes was weaker than that on the other two types of genes. Conversely, in some individuals, another pattern was observed. In WDR cv. LA1331, the average ratios in the group with three-gene copies were lower than that in the other two groups, a phenomenon also present in WDR cv. LA2951, SLL cv. LA3475, and WDR cv. LA0247. This suggests that the selection pressure on the three-copy genes is stronger compared to the other two types across these species. In WDR cv. LA0716, which contains only single-copy genes, neither two-copy nor three-copy genes were present; the ratios of *UGT* genes in this species remain below 1.

### Expression analysis and subcellular localization of *UGT* genes

To investigate the expression differences of *UGT* genes in tomato species, the RNA-seq data of SLL cv. MicroTom were analyzed, covering 6 tissues (root, stem, leaf, flower bud, flower, and fruit) and 11 stages (30, 45, 55, 65, 75, 80, 85, 88, 92, 95, and 100 days). For each tissue and stage, 3 biological replicates were included, resulting in a total of 60 samples (Table S6). The transcriptomic profile across these samples was generated based on the tomato genome (Fig. S4). Gene expression levels were quantified based on FPKM values (Table S7).

Transcriptome analysis revealed that the highly expressed protein set includes SolyMTch04g128340, SolyMTch01g036810, and SolyMTch01g042130, all of which exhibited elevated expression levels across various tissues. In contrast, certain proteins displayed tissue-specific high expression, such as SolyMTch01g042140, which was expressed in the fruit, and SolyMTch03g084750 and SolyMTch03g087150, which exhibited high expression in the root. In the flower, SolyMTch04g126030 showed high expression, whereas the expression levels of other tissues were generally low. The SolyMTch02g056010 protein displays a temporal expression pattern, characterized by low expression in early tomato development and increased expression in later stages. For example, in the stem, its expression peaked at 85 days and was nearly undetectable at 30 days. Similarly, in the fruit, the expression level was lowest at 55 days but increased significantly at 100 days. Additionally, some proteins showed consistently low expression across all tissues, e.g. SolyMTch04g139420, SolyMTch04g127870, and SolyMTch01g015610. To investigate the expression patterns of collinear genes in the MicroTom cultivar, protein sequences from the WGT collinear regions were integrated with transcriptomic data of MicroTom. Only 33 proteins were successfully matched to the transcriptome (Fig. S5). After normalization of the expression data, no consistent expression patterns were observed across different samples or conditions, suggesting possible divergence in regulatory mechanisms or transcriptional silencing of these collinear genes in MicroTom.

Copy number variation resulting from a whole-genome duplication (WGD) event can induce dosage effects in specific *UGT* genes. For example, a *UGT72B* subfamily gene, present in two protein copies (SolyMTch10g291280 and SolyMTch01g036810), exhibited significantly elevated expression across all tissues except for fruit. Similarly, a *UGT89B* subfamily gene with two protein copies (SolyMTch03g109730 and SolyMTch05g159150) were expressed in all tissues, with the highest expression observed in the fruit. In rapeseed under drought stress, a *UGT89B* subfamily member demonstrated increased transcription levels during late-stage flavonoid biosynthesis [34]. In contrast, the member of the *UGT72* subfamily with three copies (SolyMTch04g128340, SolyMTch02g067730, and SolyMTch02g075060) exhibits the highest expression in root and relatively lower expression in other tissues. Previous studies indicate that a UGT72 subfamily gene, expanded due to WGD in *Pyrus* [35], contributes to metabolic diversification. In pea, it was involved in the biosynthesis of flavonoid glucosides in seed. The *OsUGT72F1* gene *was* closely associated with heat tolerance in rice [36, 37].

Subcellular localization prediction of the *UGT* family members in tomato revealed that the majority of sequences are localized in the chloroplast (46%, 4949/10769) (Table S8). Among the 21 subfamilies, members of 16 subfamilies are predominantly localized in the chloroplast, including *UGT85* and *UGT73* subfamilies, which are the subfamilies with the highest gene numbers. Approximately 28% of *UGT* genes (3010/10769) are predicted to be localized in the cytoplasm, such as *UGT79*, *UGT80*, *UGT91*, and *UGT92* subfamilies contributing substantially to this category. A smaller proportion (18%, 1941/10769) is predicted to localize in the nucleus, mainly consisting of members from the *UGT87* subfamily. These findings are consistent with previous reports in Phoebe species [38], where a similar pattern of chloroplast, cytoplasmic, and nuclear localization was observed among *UGT* family members, suggesting a conserved subcellular distribution across different plant lineages.

### Functional divergence of *UGT* genes in *UGT73* and *UGT85* subfamilies

**Figure 5:**
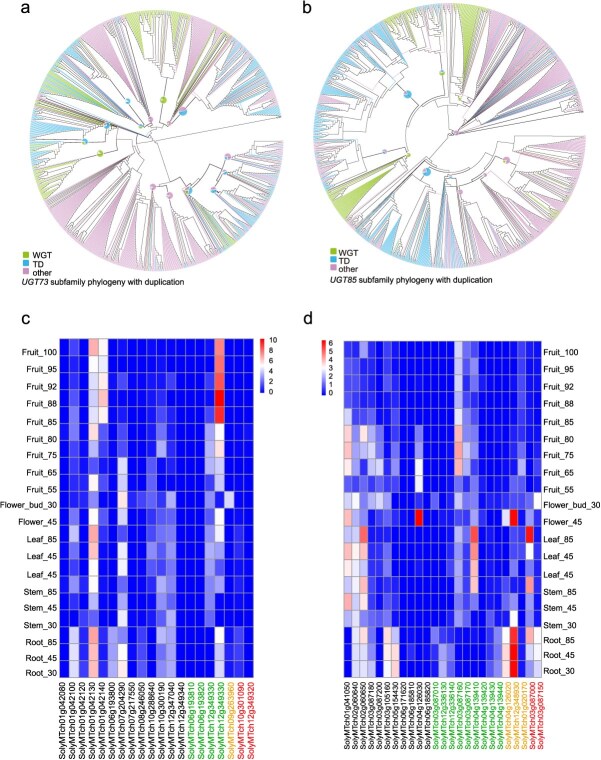
Phylogenetic trees and expression of the *UGT73* and UGT85 subfamilies. (a) phylogenetic trees of the *UGT73* subfamilies. The pie charts at the branch clade represent the proportion of different types of genes. (b) phylogenetic trees of the *UGT85* subfamilies. (c) Expression of the *UGT73* subfamily in different tissues and developmental stages of SLL cv. MicroTom, with expression levels log transformed as log₂ (1 + *x*), where *x* represents the expression level. (d) Expression of the UGT85 subfamily.

Phylogenetic analysis revealed a significant expansion of the *UGT73* and *UGT85* subfamilies. To investigate the driving forces underlying the expansion, a phylogenetic tree was constructed using algal *UGT* genes as the outgroup. The results showed that among the 1123 *UGT* genes in the *UGT73* subfamily, 213 of total members of the *UGT73* subfamily underwent a WGT event, while 311 experienced TD events (Fig. 5a). Similarly, among the 1322 *UGT* genes in the *UGT85* subfamily, 209 underwent a WGT event, and 413 experienced TD events (Fig. 5b). Additionally, in the *UGT73* subfamily, a WGT event occurred in two independent clades, whereas in the *UGT85* subfamily, a WGT event was observed in three clades. In contrast, *UGT* genes that underwent TD events were distributed across multiple clades, exhibiting a more dispersed pattern.

In the *UGT73* subfamily, the proportions of *UGT* genes that have undergone tandem duplication (TD) events in different tomato groups were as follows: 14.94% in WDR and 23.56% in SP, and the highest proportion in SLC was 25.73%, while in SLL, the proportion was 22.06%. In contrast, the proportions of genes that have undergone WGT event in these groups were 22.82%, 19.90%, 19.42%, and 16.50%, respectively. Similarly, in the UGT85 subfamily, the proportions of *UGT* genes that experienced TD events were 45.70% in WDR, 28.18% in SP, 34.15% in SLC, and 25.00% in SLL, while the proportions of *UGT* genes that experienced a WGT event were 18.36%, 16.82%, 16.67%, and 14.00%, respectively. These findings suggest that the driving forces underlying gene expansion differ across subfamilies and tomato groups. In the *UGT73* subfamily, gene expansion in SLC was largely attributed to TD events, whereas a WGT event accounts for the major proportion of gene expansion in WDR. In contrast, in the *UGT85* subfamily, both TD and WGT events were responsible for the expansion of *UGT* genes in the WDR group.

The impact of family on expression was highly significant (*F* = 5.793, *P* = 2.06 × 10^−13^), demonstrating that family membership plays a critical role in the variation of expression levels, especially for the *UGT73* family. The *UGT73* subfamilies exhibit a significantly higher number of gene copies compared to other subfamilies following WGT and TD events. Additionally, within the *UGT* pan-gene family, the proportions vary across different groups, highlighting the distinct characteristics of these two subfamilies. In the *UGT73* subfamily (Fig. 5c), the protein SolyMTch10g301100 exhibits the highest expression level, increasing expression from stem (expression value, 0.04 in 30 days) to fruit (expression value, 3170 in 88 days). This protein exhibits low expression in stem and flower bud but shows higher expression in other tissues. Its expression in fruit increases progressively during development, and this gene has undergone a TD event. In contrast, SolyMTch09g263960, which was specifically expressed in flower bud, has experienced a WGT event and exists as two copies. Other genes that have undergone either TD or WGT events, e.g. SolyMTch06g193810, SolyMTch06g193820, SolyMTch10g301090, do not exhibit a distinct expression pattern. Another highly expressed protein, SolyMTch01g042130, shows an expression increase from fruit (expression value, 2.36 in 60 days) to fruit (expression value, 273.50 in 100 days); its expression in stem progressively increases over developmental stages. Additionally, SolyMTch01g042140 was specifically expressed in fruit, with expression levels rising after 80 days of fruit development. Similarly, SolyMTch06g193800 exhibits root-specific expression.

As the subfamily with the greatest number of genes, the expression patterns of the *UGT85* subfamily are discussed in detail (Fig. 5d). The protein SolyMTch12g348930 exhibits the highest expression levels, with tissue-specific expression in root and flower. This gene has undergone a WGT event and exists as one copy. Likewise, SolyMTch04g126020, another single-copy gene, was also specifically expressed in root and flowers. However, the expression patterns of the other three one-copy genes vary: SolyMTch03g087150 was expressed in root and flower bud; SolyMTch03g087000 was expressed in root, stem, and leaf; whereas SolyMTch01g020170 exhibits consistently low expression across all tissues.

## Discussion

The observed characteristics of the *UGT* gene family provide valuable insights into the challenges and strategies in identifying *UGT* genes. Given that only the *UGT* domain is highly conserved while regions outside this domain lack conservation, it is not surprising that partial UGTs, particularly those lacking non-UGT domain regions, were identified. These shorter UGTs may represent functional variants or incomplete annotations, highlighting the complexity of *UGT* gene prediction. In our study, to address the potential limitations of standard identification methods, InterProScan was employed as an additional validation step. This ensured that all identified genes could be confidently classified as UGTs based on domain-level conservation. By excluding genes that failed this stringent validation, we minimized the risk of including erroneous or unrelated genes. This approach underscores the importance of combining bioinformatics tools with domain-specific knowledge to achieve a more accurate and comprehensive characterization of gene families, such as the *UGT* family.

The distribution and variation in the number of *UGT* subfamilies in tomato reflect the functional divergence and evolutionary adaptation of this gene family among species. The *UGT73* and *UGT85* subfamilies show relatively high gene numbers in tomato, potentially reflecting their involvement in tomato metabolism, such as contributing to the synthesis or regulation of secondary metabolites. Additionally, the *UGT82* subfamily was represented by only a single gene in tomato, indicating that its function may be highly specific or tightly regulated. These substantial differences in subfamily numbers suggest that the evolution of the *UGT* gene family has been driven by species-specific selective pressures to adapt to the ecological and physiological needs of tomato. Functional divergence, achieved through gene expansion, loss, or mutations, enables distinct subfamilies to play unique roles in tomato metabolic pathways. This distribution pattern provides valuable insights into the molecular mechanisms underlying tomato metabolism and evolutionary adaptation.

The importance and functional complexity of the *UGT85* and *UGT73* subfamilies in plants can be explained by their large number of members and their position in the phylogenetic tree. These two subfamilies have the highest number of members. *UGT85* was located at the root of the phylogenetic tree, forming a large clade with the 76 members, indicating their early divergence and expansion in evolutionary history. As major members of core OGGs, they likely play a crucial role in plant metabolism, particularly in the synthesis and regulation of secondary metabolites. This number of OGGs was inconsistent with that of many genes, indicating the presence of significant PAV in *UGT* genes within the tomato genome. Furthermore, the high representation of *UGT85* and *UGT73* in duplication events, such as TD and WGT, suggests that these subfamilies have undergone significant gene expansion. Gene expansion, likely through gene duplication or whole genome triplication mechanisms, has allowed these subfamilies to diversify their functions in specific metabolic pathways. This phenomenon further implies that *UGT85* and *UGT73* play key roles in the metabolic adaptation and response to environmental pressures in plants. The gene expansion has provided these subfamilies with enhanced metabolic regulatory capabilities, increasing the plant’s adaptability to environmental changes. These findings offer valuable insights into the molecular mechanisms underlying the diversity and adaptability of the *UGT* gene family in plant evolution.

In further studies, the functions of the *UGT85* and *UGT73* subfamilies have been validated in various plant species, such as *A. thaliana* and tobacco [9, 10]. In *A. thaliana*, the ectopic expression of *UGT85A5* enhanced the plant’s tolerance to salt stress, indicating that the *UGT85* subfamily may improve stress resistance by regulating plant metabolic pathways under adverse conditions. Similarly, *UGT73B3* and *UGT73B5* are crucial for resistance to *Pseudomonas syringae*. The expression of these two genes plays a key role in the plant’s immune response to pathogens. By regulating the synthesis of secondary metabolites and defense mechanisms, these genes enhance the plant’s defense against pathogen attacks. Therefore, these studies further validate the importance of the *UGT85* and *UGT73* subfamilies in plant metabolic regulation, stress resistance, and immune responses, supporting their crucial roles in plant evolution and adaptation.

In the *Solanum* genus, tomato underwent a WGT event, which resulted in the retention of some three copy genes in tomato compared to *V. vinifera*, while some three copy genes were lost during evolution, leaving only single-copy or double-copy genes detectable. After classifying the genes in tomato, we found that a large proportion of the three copy genes belong to the *UGT72* subfamily. This suggests that the *UGT72* subfamily may have undergone significant gene expansion and retention after the whole genome triplication event. This phenomenon likely indicates the importance of the *UGT72* subfamily in plant metabolic processes and its potential role in enhancing the plant’s ability to adapt to different environmental conditions during evolution. The *UGT72* subfamily was the highest expressed subfamily and has been found to be associated with heat tolerance in both rice and *A. thaliana* [36]. The expansion of the *UGT* family, especially the *UGT72* subfamily, may contribute to increasing metabolic diversity in plants, providing greater regulatory capacity for growth and development under various conditions. This phenomenon also suggests that the *UGT72* subfamily plays an important role in secondary metabolite biosynthesis and environmental adaptation in tomato. The large number of tomato-specific genes and their clustering at the terminal branches of the *UGT* phylogenetic tree suggest a significant degree of specialization within the tomato lineage. This distinct positioning and the extensive presence of these genes may indicate their crucial role in the unique biological functions or adaptations of tomato, reflecting the evolutionary divergence and functional specialization of the *UGT* gene family in this species.

In conclusion, we identified 10 769 *UGT* genes across 61 tomato genomes and explored their evolutionary history through phylogenetic analysis. Combined with pan-gene family analysis, we revealed the distribution of different subfamilies within various OGGs. The analysis of gene duplication events provided insights into the driving forces behind gene expansion and highlighted the characteristics of the *UGT73* and *UGT85* subfamilies. Expression analysis further revealed the tissue-specific expression of *UGT* genes and clarified the specific expression patterns of *UGT* genes that have undergone TD and WGT events across different tissues. This analysis will provide a genic resource for the study of functional *UGT* genes, paving the way for the genome-assisted breeding in the genus *Solanum*.

## Materials and methods

### Data sources

The annotated protein data of 61 tomato genomes were downloaded from http://varnatech.cn/tomatoPan/. *Amborella trichopoda*, *V. vinifera*, *A. thaliana*, and *S. tuberosum* were obtained from https://plants.ensembl.org, https://phytozome-next.jgi.doe.gov/, http://www.arabidopsis.org, and http://spuddb.uga.edu/, respectively. *Adiantum capillus-veneris* (GCA_014529385.2), *C. variabilis* (GCA_000147415.1), and *P. patens* (GCF_000002425.4) data were downloaded from a public database: the National Centre for Biotechnology Information (NCBI; http://www.ncbi.nlm.nih.gov/). *Cycas panzhihuaensis* data were downloaded from https://db.cngb.org/codeplot/datasets/public_dataset?id=PwRftGHfPs5qG3gE. *Ginkgo biloba* data were downloaded from the Ginkgo Database (Ginkgo Database—Genome (zju.edu.cn)). The Hidden Markov Model (HMM) profile of the UDP-glycosyltransferase domain (PF00201) was retrieved from Pfam 29.0 (Browse—InterPro (ebi.ac.uk)).

The 61 tomato species were divided into four main groups based on their domestication history: the wild distant relatives (WDR), the ancestral species (SP), the early domesticated group (SLC), and the cultivated group (SLL).

### Identification of UDP-glycosyltransferase proteins

Putative *UGT* proteins were identified following the method described by Yu *et al.* [39]. The HMM profile of the *UGT* domain (PF00201) was employed to screen the genomes of various species. In summary, UGT genes were detected using the HMMER [40] program with the ‘—cut_tc’ threshold. High-confidence *UGT* protein sequences were then selected from the putative *UGT* genes to perform multiple sequence alignments using MAFFT [41]. Then, these highly conserved sequences were subsequently utilized to construct species-specific HMM profiles via the ‘hmmbuild’ module of the HMMER program. These species-specific HMM profiles were applied to classify the final *UGT* gene sets from the genomic data of the aforementioned plant species using the HMMER program with a threshold of *E* > 0.01. Finally, InterProScan [42] was used to validate the identified *UGT* genes. Orthologous *UGT* gene clusters in the 61 tomato accessions were inferred using the OrthoFinder software.

### Family classification and phylogenetic analysis of *UGT* genes

The classification of *UGT* genes was determined using system clustering against the *A. thaliana UGT* gene family database (*UGT* Gene Names | Washington State University [wsu.edu]), which was organized into 21 UGT gene families based on previous studies. Phylogenetic analysis reveals that members of the same UGT gene families cluster together. The final UGT protein sequence datasets were used to perform multiple sequence alignments using the MAFFT program with default settings. To ensure conservation, the alignment data were trimmed using the ‘gappyout’ parameter in the TrimAl software [43]. Subsequently, the trimmed sequences were utilized to construct phylogenetic trees using the maximum likelihood method in IQ-TREE [44], with 1000 bootstrap replicates.

### Collinear analysis in tomato species compared to *Vitis vinifera*

The MCScanX toolkit [45] was employed to identify putative homologous chromosomal regions between tomato genomes and *V. vinifera* genomes with the following parameters: *e* = 1 × 10–20, *u* = 1, and *s* = 5. According to the collinear analysis procedures using MCScan algorithm, high similarity genes were recognized as homologous regions between multiple genomes, which can help us identify orthologous gene pairs in tomato species compared with *V. vinifera* genomes.

### Identification and analysis of tandemly duplicated *UGT* genes

Tandemly duplicated genes were identified using a structured approach. GFF files were filtered for mRNA entries and sorted by genomic position. Protein sequences underwent BLASTP analysis to identify candidate gene pairs, with an *E*-value threshold of 1e−20. Gene distances were calculated and subjected to hierarchical clustering to identify gene clusters. BLAST results were processed to facilitate direct gene comparisons. Clustering results were filtered to exclude alternative splicing variants, ensuring unique gene identification. Final gene lists were generated for each genome, highlighting the tandemly duplicated genes identified through this analysis.

### Selection pressures of *UGT* genes

The ratios of the rates of nonsynonymous to synonymous substitutions (*K*a/*K*s) of *UGT* gene pairs between tomato and *V. vinifera* were calculated to estimate selection modes by using PAML software. The *K*a/*K*s ratios greater than 1, less than 1, and equal to 1 represent positive selection, negative selection, and neutral selection, respectively.

### Expression profiling and subcellular localization prediction of *UGT* genes in tomato

The expression values for each *UGT* gene were calculated by fragments per kilobase of the exon model per million mapped reads by using the RNA-seq data of tomato from the GEO database (CRA001723). The Spearman rank correlation was used to calculate the distance matrix between the gene expression data of *UGT* in tomato. Further, the complete linkage clustering was used for hierarchical clustering of *UGT* genes in tomato. Expression heat maps of *UGT* genes in tomato were generated by R language packages.

Subcellular localization prediction was performed using WoLF PSORT [46] software with the plant model and default parameters. All UGT protein sequences were submitted to the WoLF PSORT program, which applies a k-nearest neighbor (k-NN) classification algorithm to predict protein localization. For each protein, predicted subcellular locations were ranked based on their scores, with higher scores indicating a greater likelihood of localization. The top-scoring location was considered the primary predicted localization site. The distribution of predicted localizations was subsequently summarized and compared among different *UGT* subfamilies.

## Supplementary Material

Web_Material_uhaf204

## Data Availability

All the data were obtained from public databases, with detailed information provided in the Materials and methods.
