# Supplementary material for: Evolutionary dynamics and functional divergence of the UDP-glycosyltransferases gene family revealed by a pangenome-wide analysis in tomato
Source: Hortic Res. 2025 Jul 21;12(11):uhaf204. doi: 10.1093/hr/uhaf204 (PMC12574540; doi:10.1093/hr/uhaf204)
Supplement: Web_Material_uhaf204 [file web_material_uhaf204.zip › FigureS5.pdf]

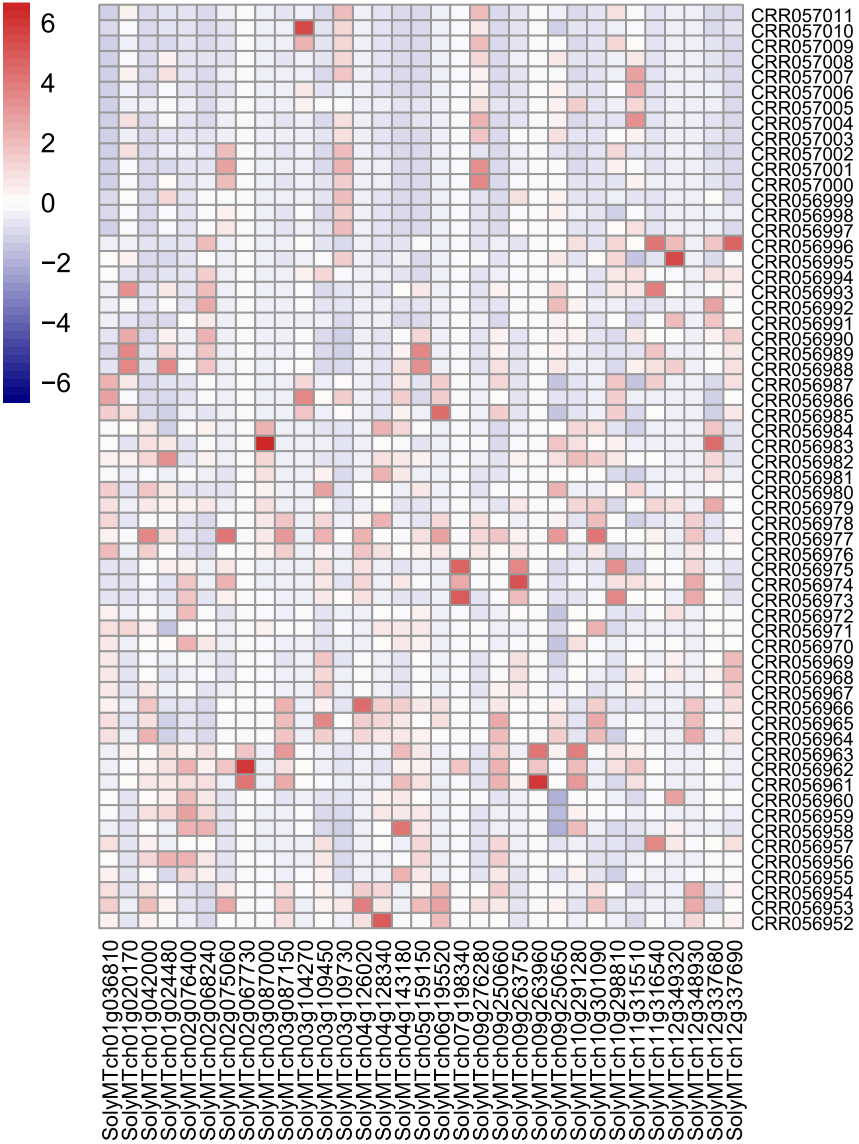
6
4
2
0
−2
−4
−6
CRR057011
CRR057010
CRR057009
CRR057008
CRR057007
CRR057006
CRR057005
CRR057004
CRR057003
CRR057002
CRR057001
CRR057000
CRR056999
CRR056998
CRR056997
CRR056996
CRR056995
CRR056994
CRR056993
CRR056992
CRR056991
CRR056990
CRR056989
CRR056988
CRR056987
CRR056986
CRR056985
CRR056984
CRR056983
CRR056982
CRR056981
CRR056980
CRR056979
CRR056978
CRR056977
CRR056976
CRR056975
CRR056974
CRR056973
CRR056972
CRR056971
CRR056970
CRR056969
CRR056968
CRR056967
CRR056966
CRR056965
CRR056964
CRR056963
CRR056962
CRR056961
CRR056960
CRR056959
CRR056958
CRR056957
CRR056956
CRR056955
CRR056954
CRR056953
CRR056952
SolyMTch01g036810
SolyMTch01g020170
SolyMTch01g042000
SolyMTch01g024480
SolyMTch02g076400
SolyMTch02g068240
SolyMTch02g075060
SolyMTch02g067730
SolyMTch03g087000
SolyMTch03g087150
SolyMTch03g104270
SolyMTch03g109450
SolyMTch03g109730
SolyMTch04g126020
SolyMTch04g128340
SolyMTch04g143180
SolyMTch05g159150
SolyMTch06g195520
SolyMTch07g198340
SolyMTch09g276280
SolyMTch09g250660
SolyMTch09g263750
SolyMTch09g263960
SolyMTch09g250650
SolyMTch10g291280
SolyMTch10g301090
SolyMTch10g298810
SolyMTch11g315510
SolyMTch11g316540
SolyMTch12g349320
SolyMTch12g348930
SolyMTch12g337680
SolyMTch12g337690
