# Supplementary figures and images for: Evolutionary dynamics and functional divergence of the UDP-glycosyltransferases gene family revealed by a pangenome-wide analysis in tomato
Source: Hortic Res. 2025 Jul 21;12(11):uhaf204. doi: 10.1093/hr/uhaf204 (PMC12574540; doi:10.1093/hr/uhaf204)

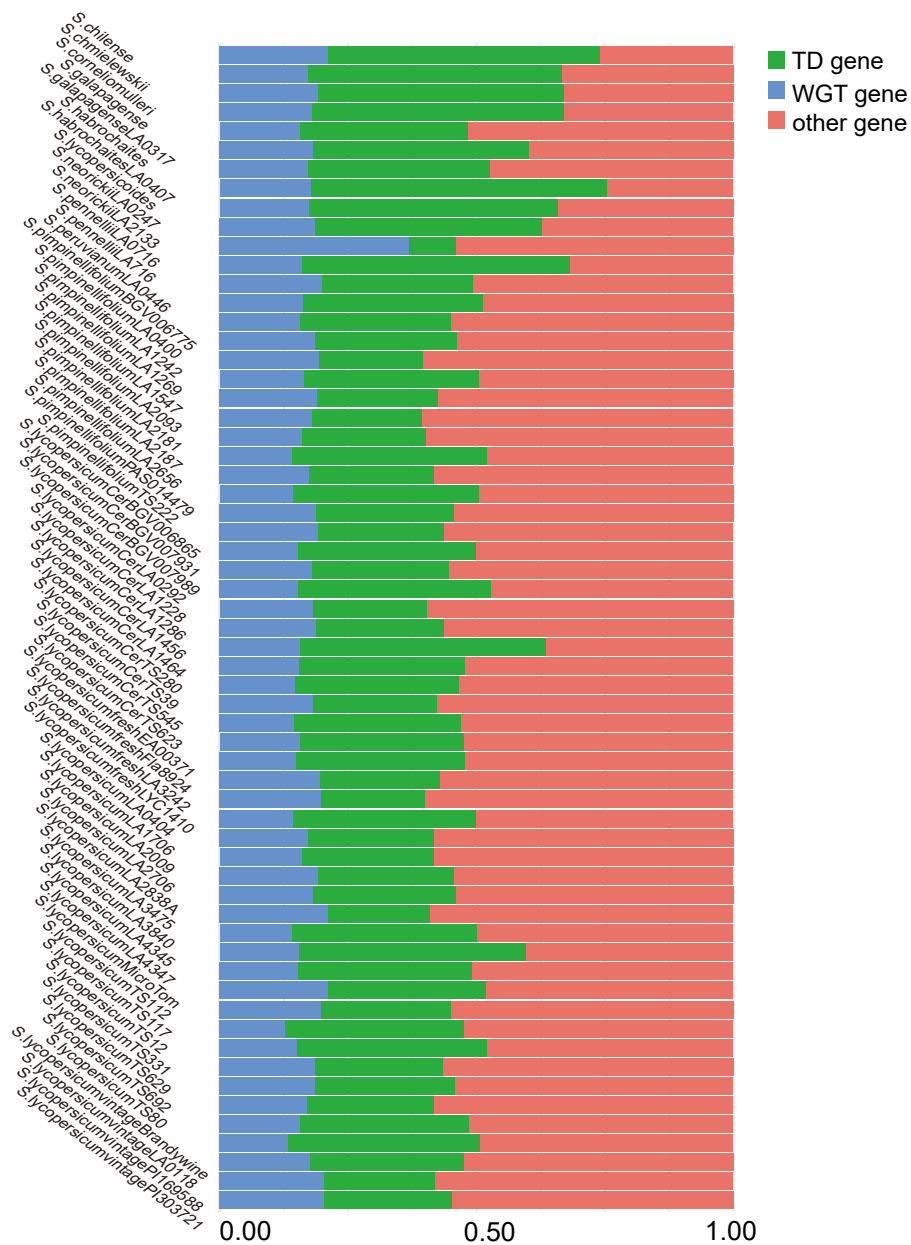

Supplement: Web_Material_uhaf204 [file web_material_uhaf204.zip › FigureS1.pdf]

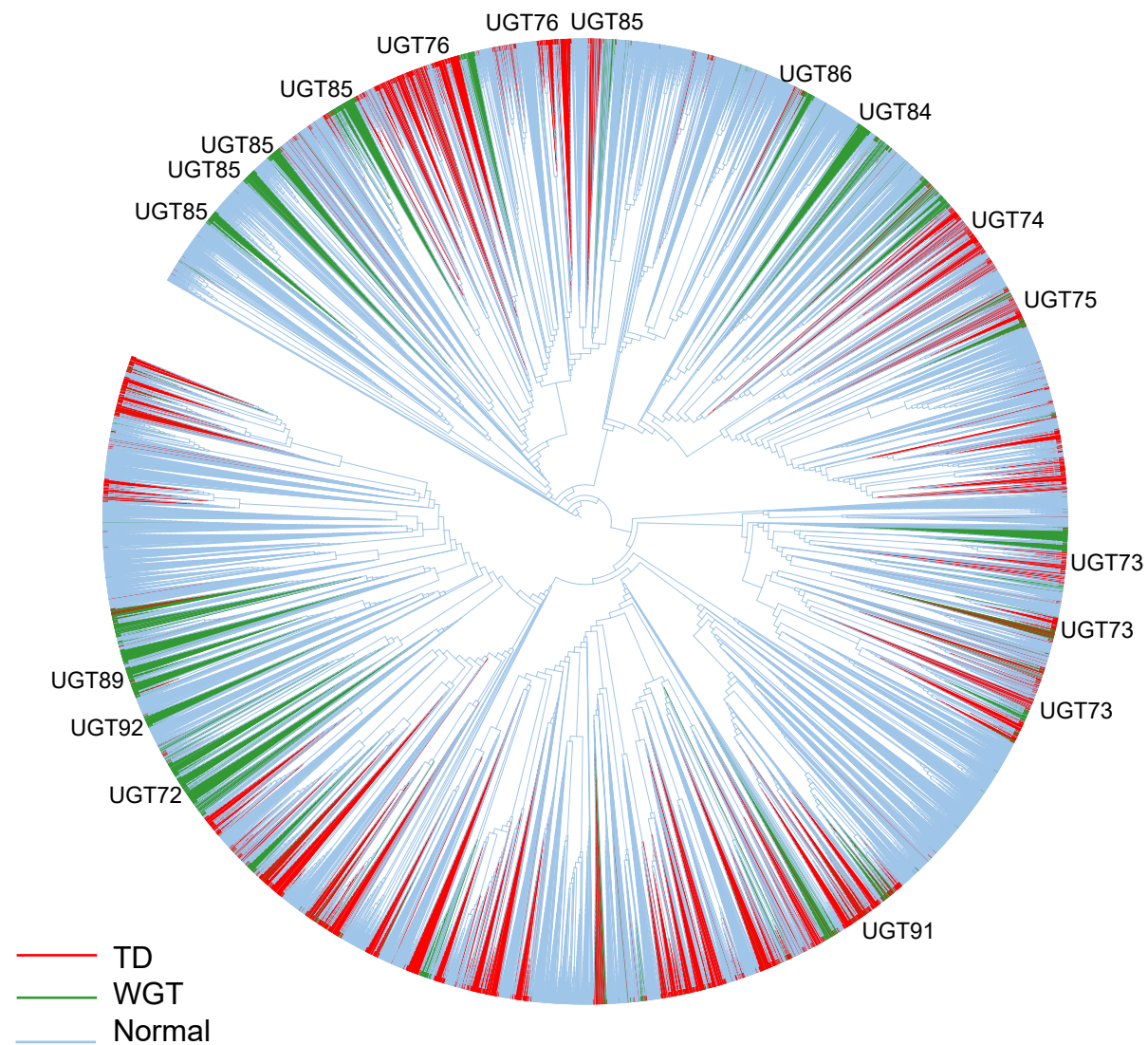

UGT gene family with duplication

Supplement: Web_Material_uhaf204 [file web_material_uhaf204.zip › FigureS2.pdf]

Comparison of omega values

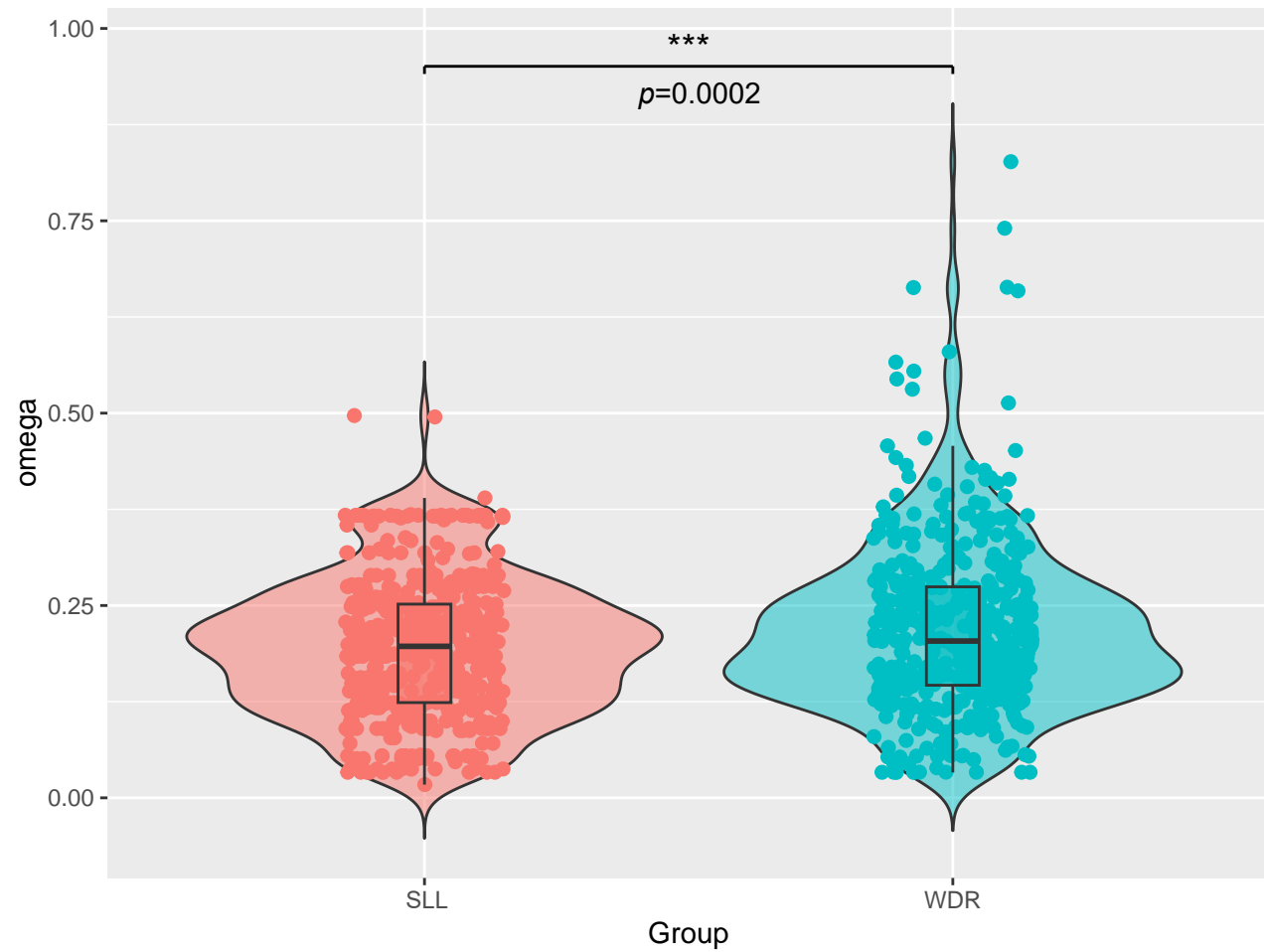

Supplement: Web_Material_uhaf204 [file web_material_uhaf204.zip › FigureS3.pdf]

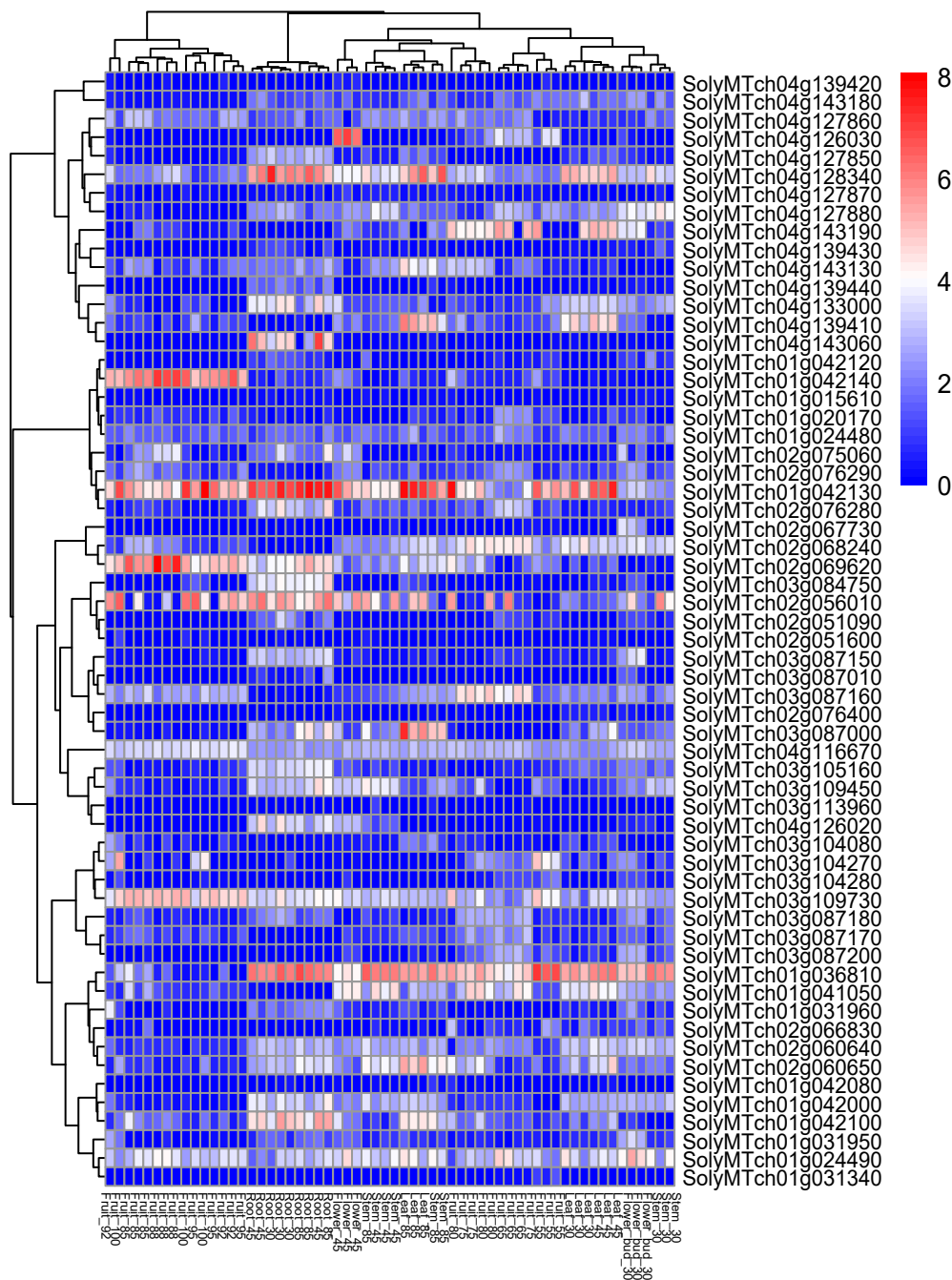

Supplement: Web_Material_uhaf204 [file web_material_uhaf204.zip › FigureS4.pdf]
